# The Story of the Insane from Year to Year

**Published:** 1900-05-05

**Authors:** 


### THE STORY OF THE INSANE FROM YEAR TO YEAR.

Twelfth Series.

### THE GARTLOCH ASYLUM, GLASGOW.

This asylum has issued its second annual report, from which we learn it had in residence 454 patients. During the year there were 140 admissions, 63 readmissions, 98 recoveries, 79 discharged relieved, and 38 deaths. Of these deaths 19 were caused by cerebral and spinal diseases, 4 by consumption, 1 by pneumonia, and 1 by enteritis. Of the admissions 50 were driven insane by intemperance in drink, 9 by hereditary tendency, and 8 by previous attacks. Calculated in the usual way the recoveries stand at 48 per cent., and the deaths at 8 per cent. The first is considerably above the average, and the latter slightly below it. Dr. Oswald tells us that 44 cases were boarded out during the year, and that only seven were returned to the asylum. Could this sensible plan of dealing with the insane be transplanted to England it would make an enormous difference to the population of our county asylums, but for some reason or other it has never taken root with us. Possibly the favourable conditions present in Scotland are absent in England, or it may be that no one having sufficient authority will take the initiative. It speaks well for the management of this asylum that 25 per cent. of the patients are on parole and are allowed to walk about the grounds or beyond them as they please. In only seven instances was the privilege abused. As far as our knowledge of English asylums extends there is not one in which anything approaching 25 per cent. are on parole, and we regret that it is so; but here again, we suppose, the bold initiative has not been taken. The Commissioners say that the nursing of the sick is well organised and efficiently conducted, and the committee record their "high appreciation of the services of Dr. Oswald and his staff."

### LANCASHIRE COUNTY ASYLUM AT PRESTWICH.

This is, we believe, the largest asylum in England, and on December 31st, 1898, it contained 2,670 patients, having added 31 to its numbers during the year. There were 325 first admissions, 73 readmissions, 185 recoveries, 48 discharged relieved, and 133 deaths. The recoveries stand at the very creditable percentage of 46·95 on the admissions, and the deaths are extremely low, being only 5 per cent. of the average number resident. Of the deaths 58 were caused by cerebral and spinal diseases, 8 by pneumonia, 24 by consumption, and 4 by bronchitis. Various moral causes account for the insanity in 131 of the admissions, intemperance in drink for 104, and hereditary influence for 86. Mr. Ley says the asylum has been full during the whole of the year, and he thinks great care should be exercised by the Union authorities in selecting such cases as really need asylum treatment to fill such vacancies as arise from time to time. This is true enough from the asylum standpoint, but from the Union standpoint it has no force at all, because sufficient accommodation ought to have been long since provided for the county, and the guardians are quite justified in sending every insane person to the county asylum. If the Asylum Committee declines to meet the demands of the guardians it would be rather hard to make the latter suffer for the shortcomings of the former. Mr. Ley further tells us that the asylum has been remarkably free from contagious or infectious diseases, and that the sanitary condition has been satisfactory, "notwithstanding some adverse remarks in the Commissioners in Lunacy's last report to the Lord Chancellor." It does not appear that his Lordship has taken any notice of the report, whatever it was, and the Committee of Visitors say it is a "regrettable fallacy." Post-mortem examinations were made in 80 per cent. of the deaths. The assistant medical officers continue their lectures and instructions to the staff, and the "aim has been to perfect a staff of experienced attendants, imbued with an intelligent appreciation of their responsibilities and duties." The weekly rate of maintenance was 8s. 4¾d. per head.

### NORTHUMBERLAND COUNTY ASYLUM, MORPETH.

In this asylum we note an increase from 595 to 618 during the year 1898. There were 143 first admissions, 58 readmissions, 80 recoveries, and 83 deaths. The recoveries stand at the very creditable percentage of 44·6 on the admissions of the year; but it should be noticed that the number of readmissions was also very high. The deaths were also high, being 13·4 on the average number resident. Of these deaths, 28 were caused by cerebral and spinal diseases, 30 by consumption, 4 by general tuberculosis, and 2 by pneumonia. Of the year's admissions, 11 owed their insanity to various moral causes, 32 to intemperance in drink, and 19 to hereditary influences. In 57 the cause was unknown, and such a high proportion of unknowns must weaken the value of Table X. When speaking of the death rate and the high mortality from consumption, Dr. McDowall says that close attention to the subject made it appear to him "almost, if not quite, certain that tubercular infection was contracted almost exclusively in the dormitories where direct cross-ventilation did not exist." We are pleased to quote this opinion from one of the most experienced medical superintendents in England, and it corroborates the view we lately expressed when noticing the report of another asylum. We are also of opinion that the evils of this absence of direct cross-ventilation are greatly intensified if the rooms be warmed by any system of hot-air or hot-water apparatus. We should add that structural alterations have been made at the Morpeth Asylum whereby the desired ventilation has been obtained. We are pleased to note that villa residences for private patients are being erected. Northumberland here gives a lead which we hope other counties will soon follow. The visitors say they "have pleasure in testifying to the general efficiency of the medical superintendent." The weekly cost of maintenance is a fraction over 10s. a head.

### THE RETREAT, YORK.

Verily this asylum is no *parvenu*, for it now issues its one hundred and third annual report. At the beginning of the year it contained 167 patients, and at the end the number had fallen slightly. The year referred to comprises the period between July 1st, 1898, and June 30th, 1899.

There were 23 first admissions, 14 readmissions, 11 recoveries, and 11 deaths. The recoveries stand at 29·72 per cent. of the admissions, and the deaths at 6·63 per cent. of the average number resident. The recovery rate is considerably below the average at the Retreat, which, since the opening of the asylum in 1796, stands at 40·39 for all classes; but if the "Friends" only be taken into consideration the average is 43·61. Why the "Friends" should have this high percentage, and the non-"Friends" only 34·34 per cent. through a long series of years, is not very apparent. On the other hand the death-rate is 5·48 per cent. among the "Friends" and only 4·89 per cent. among the non-"Friends." Dr. Pierce thinks it will soon be necessary to consider what steps should be taken to decrease the number of chronic cases so as to admit more acute cases; but he does not at present suggest how this is to be done, and he admits there is "a distinct duty towards the unfortunate patient who has permanently broken down." We are of course aware that some of the lunatic hospitals have adopted this turning-out process, to the great inconvenience of the patient and the patient's friends, but we hardly expected to find such a step even hinted at in the Retreat at York. During the year a block for the accommodation of eight nurses was opened, and probationer nurses are now received for a three years' course of training. The charges for patients vary from £2 2s. a week to £7 7s. a week, but members of the society are received at lower rates if necessity requires. There are two assistant medical officers, and one of these is a lady.

### THE EASTERN COUNTIES' ASYLUM FOR IDIOTS, COLCHESTER.

On January 1st, 1898, this asylum contained 251 patients, and, practically, the numbers were stationary during the year. There were 33 admissions, 14 discharges, and 20 deaths. As usual, Mr. Turner's report gives us a plain, straightforward account of the year's history, and it is very pleasing to notice that so much can be done to improve the condition of those poor little waifs that find their way into our idiot asylums. From personal inspection we are able to speak highly of the homeliness of the surroundings which the children possess at the Colchester Asylum, and we learn that 50 per cent. of the discharges during 1898 had improved so much as to be of use in various ways. Beyond a doubt, if these cases had not been placed under treatment they would have gone from bad to worse and ended in a perfectly hopeless condition. The medical report is presented by Dr. Fry. It is necessarily short, as there cannot be much to relate concerning the purely medical history of 250 fairly healthy children, but we think it would be a good innovation if Dr. Fry were to, in his next report, classify the idiots according to types, tell us which type is the most educable, and if possible tell us, after careful inquiries, what was the probable cause of the idiocy in the year's admissions. Of the 20 deaths we find that, as is usual in idiot asylums, consumption is the disease that kills the greatest number. The asylum is to be immensely improved by the erection of new class-rooms and workshops, and these are to be known as the "Peckover Schools," from the name of the donor. The foundation-stone was laid by Mr. Peckover in March, 1898, so that by this time it is probable the building is finished and occupied. The report says the building is to be heated throughout by hot water. If this means that the hot water is to be used as an auxiliary to open fireplaces, it is valuable in certain conditions; but if it means that open fireplaces are to be abolished, and that the patients are to inhale this artificially warmed air, it is bad in principle and in practice is sure to increase the prevalence of consumption. During the year the income exceeded the expenditure, and it was decided to place £622 to the reserve fund. About £1,500 are now wanted to build a connecting corridor, gateway, show-rooms, and porter's lodge; and we hope we shall next year be able to chronicle that some benevolent man or men will have followed Mr. Peckover's lead. The average weekly cost of maintenance was 12s. a head, and this sum included establishment charges.

